# *OsWRKY74*, a WRKY transcription factor, modulates tolerance to phosphate starvation in rice

**DOI:** 10.1093/jxb/erv515

**Published:** 2015-12-11

**Authors:** Xiaoyan Dai, Yuanyuan Wang, Wen-Hao Zhang

**Affiliations:** State Key Laboratory of Vegetation and Environmental Change, Institute of Botany, Chinese Academy of Sciences, Beijing 100093, China

**Keywords:** *OsWRKY74*, phosphate starvation, rice (*Oryza sativa*), root system architecture, transgenic.

## Abstract

The WRKY transcription factor family has 109 members in the rice genome, and has been reported to be involved in the regulation of biotic and abiotic stress in plants. Here, we demonstrated that a rice OsWRKY74 belonging to group III of the WRKY transcription factor family was involved in tolerance to phosphate (Pi) starvation. OsWRKY74 was localized in the nucleus and mainly expressed in roots and leaves. Overexpression of *OsWRKY74* significantly enhanced tolerance to Pi starvation, whereas transgenic lines with down-regulation of *OsWRKY74* were sensitive to Pi starvation. Root and shoot biomass, and phosphorus (P) concentration in rice *OsWRKY74*-overexpressing plants were ~16% higher than those of wild-type (WT) plants in Pi-deficient hydroponic solution. In soil pot experiments, >24% increases in tiller number, grain weight and P concentration were observed in rice *OsWRKY74*-overexpressing plants compared to WT plants when grown in P-deficient medium. Furthermore, Pi starvation-induced changes in root system architecture were more profound in *OsWRKY74*-overexpressing plants than in WT plants. Expression patterns of a number of Pi-responsive genes were altered in the *OsWRKY74*-overexpressing and RNA interference lines. In addition, *OsWRKY74* may also be involved in the response to deficiencies in iron (Fe) and nitrogen (N) as well as cold stress in rice. In Pi-deficient conditions, *OsWRKY74*-overexpressing plants exhibited greater accumulation of Fe and up-regulation of the cold-responsive genes than WT plants. These findings highlight the role of *OsWRKY74* in modulation of Pi homeostasis and potential crosstalk between P starvation and Fe starvation, and cold stress in rice.

## Highlight

The WRKY transcription factor family in rice is functionally diverse. We demonstrate that WRKY74 overexpression enhances growth, increases tiller number, grain weight and phosphorus concentration under phosphate-deprived conditions in rice.

## Introduction

Phosphorus (P) is one of the essential macronutrients for plant growth and development. It is a constituent of key molecules such as ATP, nucleic acids and phospholipids (Marschner, 1995; Raghothama, 1999). Plants take up P exclusively in the form of inorganic phosphate (Pi). Although the overall P content in soil is generally high, in many natural and agricultural ecosystems, plants often have to face conditions in which availability of Pi is at lower extremities (Raghothama, 1999; Richardson *et al.*, 2009; Rouached *et al.*, 2010; Hinsinger *et al.*, 2011; Plaxton and Tran, 2011). To cope with Pi deficiency, plants have evolved numerous strategies to optimize Pi acquisition from soil and its distribution to different organs and sub-cellular compartments (Raghothama 1999, 2000; Lynch, 2011; Péret *et al.*, 2011). For example, stimulation of lateral roots and root hairs leads to profound changes in root system architecture to maximize root surface area for Pi uptake under Pi-deficient conditions (Williamson *et al.*, 2001; López-Bucio *et al.*, 2003; Ticconi and Abel, 2004; Svistoonoff *et al.*, 2007). Exudation of organic anions and phosphatases, as well as acidification of the rhizosphere have been used to liberate plant available P by solubilizing Pi bounded to soil particles (Jones, 1998; Richardson *et al.*, 2009). In addition, Pi-starved plants can regulate multiple metabolic processes to reprioritize utilization of internal Pi and maximize acquisition of external Pi to adapt to low Pi environments (Vance *et al.*, 2003; Wissuwa, 2003). The complex network of regulatory genes necessary to sense and respond to Pi deficiency is being dissected (Smith *et al.*, 2010; Yang and Finnegan, 2010; Hammond and White, 2011; Kuo and Chiou, 2011). For example, in *Arabidopsis thaliana*, a major transcriptional regulatory system that involves *PHR1*, *SIZ1*, *miR399* and *PHO2* in response to Pi deficiency has been identified (Rubio *et al.*, 2001; Fujii *et al.*, 2005; Aung *et al.*, 2006; Schachtman and Shin, 2007). In contrast to Arabidopsis, recent studies have shown that the *PHR1*-*miR399*-*PHO2* signalling pathway also operates in rice plants in response to Pi deficiency. For instance, Zhou *et al.* (2008) demonstrated that *OsPHR2*, the homologue of *AtPHR1*, is a key regulator involved in Pi starvation signalling in rice. *OsSPX1* is associated with Pi homeostasis and suppresses the function of *OsPHR2* by regulating *OsPT2* expression (Wang *et al.* 2009; Liu *et al.* 2010). The *PHR1*-*miR399*-*PHO2* pathway is a central component of the Pi starvation response, but several lines of evidence demonstrate that some Pi-responsive transcriptional factors (TFs) are not involved in the *PHR1*-*miR399*-*PHO2* pathway (Yi *et al.*, 2005; Nilsson *et al.*, 2010). These include *OsPTF1* (Yi *et al.*, 2005) in rice, and *MYB62* (Devaiah *et al.*, 2009), *WRKY75* (Devaiah *et al.*, 2007a), *ZAT6* (Devaiah *et al.*, 2007b) and *BHLH32* (Chen *et al.*, 2007) in Arabidopsis. These TFs function in crosstalk between Pi-starvation signalling and signalling cascades associated with phytohormones and photosynthates, to govern physiological responses to Pi limitation (Rouached *et al.*, 2010).

WRKY TFs are a large family of regulatory proteins in plants. The most prominent feature of these proteins is the presence of the WRKY domain, which is a 60 amino acid region with a strongly conserved amino acid sequence WRKYGQK in its N-terminal, and a novel potential C-C-H-H/C zinc-finger motif in its C-terminal (Eulgem *et al.*, 2000). Based on the number of WRKY domains and the type of their zinc-finger motif, WRKY proteins are classified into three distinct groups (Eulgem *et al.*, 2000). Compared with other multigene families of plant TFs, the percentage of *WRKY* gene family members that are responsive to biotic stress is relatively high, implying that WRKY proteins may play key roles in the regulation of biotic stresses (Ulker and Somssich, 2004). For example, 49 out of 72 examined *WRKY* genes in Arabidopsis are responsive to bacterial infection or salicylic acid (SA) treatment (Dong *et al.*, 2003), and the majority of *WRKY* genes within group III of the WRKY family are responsive to both SA and pathogen infection (Kalde *et al.*, 2003). In contrast to biotic stresses, little information is known about the roles of WRKY proteins in plant responses to abiotic stress in general and Pi starvation in particular. In Arabidopsis, only four WRKY proteins, i.e. *AtWRKY75*, *AtWRKY6*, *AtWRKY45* and *AtWRKY42*, have been reported to be involved in Pi starvation (Devaiah *et al.*, 2007a, Chen *et al.*, 2009, Wang *et al.*, 2014, Su *et al.*, 2015). For example, *WRKY75* acts as a positive regulator of Pi stress responses and RNAi suppression of *WRKY75* results in impaired Pi starvation responses in Arabidopsis (Devaiah *et al.*, 2007a). *AtWRKY6* negatively regulates Pi starvation response by modulating Arabidopsis PHOSPHATE1 (*PHO1*) expression (Chen *et al.*, 2009). Moreover, *AtWRKY6* has been demonstrated to be positively involved in the regulation of response to boron deficiency (Kasajima and Fujiawara, 2007; Kasajima *et al.*, 2010). *WRKY45* regulates Pi uptake by modulating expression of *PHT1;1* in Arabidopsis (Wang *et al.*, 2014). *WRKY42*, a homologue of *WRKY6* in Arabidopsis (Eulgem *et al.*, 2000), modulates Pi homeostasis by regulating the expression of *PHO1* and *PHT1;1* (Su *et al.*, 2015).

Rice is a model monocot plant and one of the most important food crops in Asia (Cantrell and Reeves, 2002). Rice growth, development and productivity are seriously affected by Pi availability in many areas worldwide (Raghothama, 1999; Gamuyao *et al.*, 2012), but the Pi starvation signalling pathway is still largely unknown in rice. In the present study, we identified a WRKY TF belonging to group III of the WRKY family, designated *OsWRKY74*, in rice. Our results demonstrated that overexpression of *OsWRKYP74* in rice conferred the transgenic plants greater tolerance to low Pi stress by activating Pi starvation-induced genes and modulating root system architecture. In addition to the involvement of *OsWRKY74* in multiple Pi starvation responses, analysis of transgenic plants with overexpressing and RNAi *OsWRKY74* provided direct evidence that *OsWRKY74* was also involved in Fe deficiency and cold stress. These results demonstrate that *OsWRKY74* participates in the regulation of multiple nutrient starvation responses and cold stress, highlighting the possible roles of *OsWRKY74* in crosstalk between P and Fe, and P and cold stress.

## Materials and methods

### Plant materials and growth conditions

*Japonica* rice cv. Zhonghua 10 was used in physiological experiments and rice transformation throughout this study. For hydroponic culture of the seedlings, rice seeds were surface sterilized for 5min with ethanol (75%, v/v) and for 10min with commercially diluted (1:3, v/v) NaClO, followed by several rinses with sterile water. Seeds were germinated in the dark at 28°C for 72h. Thereafter seedlings were grown in a greenhouse. Then, the 7-d-old seedlings were transferred to nutrient solution containing 1.425mM NH_4_NO_3_, 0.513mM K_2_SO_4_, 0.998mM CaCl_2_, 1.643mM MgSO_4_, 0.168mM Na_2_SiO_3_, 0.125mM Fe-EDTA, 0.019mM H_3_BO_3_, 0.009mM MnCl_2_, 0.155mM CuSO_4_, 0.152mM ZnSO_4_ and 0.075mM Na_2_MoO_4_, pH 5.5, supplemented with 0.323mM NaH_2_PO_4_ (HP) or 0.016mM NaH_2_PO_4_ (LP). The hydroponic experiments were carried out in a growth room with a 16-h-light (30°C)/8-h-dark (22°C) photoperiod and the relative humidity was controlled at ~70%. The solution was refreshed every 3 d (Wang *et al.*, 2009).

To minimize recycling of P from seed endosperm, the seed endosperm was removed prior to transfer of rice seedlings to Pi-deficient medium. The optimal time and concentration used for the low-Pi stress were determined following protocols described by Liu *et al.* (2010). The concentration of Pi deficiency was set at 0.016mM throughout this study. One-week-old wild-type (WT) and transgenic rice plants were exposed to the low-Pi solution (0.016mM Pi) for 30 d or 14 d. For analyses of root system architecture and RT-PCR, rice seedlings grown in the low-Pi (0.016mM Pi) solution for 14 d were used. P concentration, shoot biomass and root biomass were measured after 30 d of Pi starvation.

To determine the effect of deprivation of other mineral nutrients, including nitrogen (N), iron (Fe), and potassium (K) on *OsWRKY74* expression, 1-week-old WT and transgenic rice plants were exposed to solution containing no nitrogen (−N), no potassium (−K), and no iron (–Fe), respectively. Plants were harvested for RNA extraction after the treatments for varying periods (0, 6 and 12h; 1, 3, 5 and 7 d).

For the treatment of salt and osmotic stress, seedlings were exposed to solution containing 200mM NaCl or 15% PEG 6000 and leaves were sampled after 5h, respectively. For cold stress, seedlings were transferred to a growth chamber at 4°C for 5, 12, 24 and 72h, and sampled for further analysis.

For pot experiments in soil, the experiments were performed in an experimental field of the Institute of Botany, Chinese Academy of Sciences. At 14 d after germination (DAG), WT and transgenic seedlings were transferred into pots with two Pi levels; 60mg Pi kg^-1^ soil as KH_2_PO_4_ and 15mg Pi kg ^-1^ soil. Each pot received the equivalent of 200 mgN kg^-1^ soil (as urea) and 130mg K_2_O kg^-1^ (as K_2_SO_4_).

### Quantitative real-time PCR

Three biological replicates, each comprising five individual plants, were used for quantitative real-time PCR. Total RNA was extracted using Trizol reagent (Invitrogen). 2 μg of total RNA was treated with DNAase I (Promega) and then transcribed in a total volume of 20 μl with 1 μg oligo (Dt)_18,_ 10mM deoxynucleotide triphosphate, and 200 units SuperScripts^TM^ II reverse transcriptase (Invitrogen). The cDNA samples were diluted to 2 and 8ng μl^-1^. Triplicate quantitative assays were performed on 1 μl of each cDNA dilution with the SYBR Green Master Mix or TaqMan reagents (TaKaRa) and an ABI 7900 sequence detection system according to the manufacturer’s protocol (Applied Biosystems). The relative quantification method (Delta-Delta cycle threshold) was used to evaluate quantitative variation between the replicates examined. The PCR signals were normalized to those of *Actin* or *rice polyubiquitin1* (*RubQ1*). All the primers used for the quantitative RT-PCR (RT-qPCR) are listed in Supplementary Tables S1, S2 at *JXB* online. Detection and quantification of mature miR399 were performed as previously described (Wang *et al.*, 2011), Briefly, RNA was reversely transcribed using One Step PrimeScript miRNA cDNA Synthesis Kit (TaKaRa). This kit adds poly (A) to the 3′ end of miRNAs and starts to reverse transcribe. The reverse transcription was led by a kind of special oligo-dT ligated with a known sequence at its 5′ end. RT-qPCR was performed using SYBR Premix Ex Tag II (TaKaRa).

### Localization of OsWRKY74-GFP fusion proteins

The whole coding sequence of *O*s*WRKY74* was amplified with two primers, 5′-GC*TCTAGA*ATGGAGAGCATGGAGGGC-3′ (*Xba*I site underlined) and 5′-CGG *GTACC*TGCGAAGAAG CTGGTGATATC-3′ (*Kpn*I site underlined). The PCR product was subcloned into the pBI221 vector to generate *pBI221- O*s*WRKY74-GFP*, containing an *O*s*WRKY74-GFP* fusion construct under the control of the CaMV 35S promoter. The construct was confirmed by sequencing and used for transient transformation of onion (*Allium cepa*) epidermis via a gene gun (Bio-Rad). Transformed onion cells were observed with a confocal microscope (Nikon).

### Plasmid construction and plant transformation

For *O*s*WRKY74* RNAi, a fragment of 337bp was amplified from *O*s*WRKY74* with two primers, 5′-GG*GGTACCACTA GT*ATGGAGAGCATGGAGGG-3′ (*Kpn* I and *Spe* I sites underlined) and 5′-CG*GGATCCGAGCTC*TAATCTGATGCCTCTTC-3′ (*Bam*H I and *Sac* I sites underlined), containing two restriction enzymes at their 5′ ends, respectively. The hairpin structure consisting of an antisense *O*s*WRKY74* fragment, a rice intron and an *O*s*WRKY74* sense fragment were inserted between the maize ubiquitin promoter and the nopaline synthase terminator of the vector pTCK303 (Supplementary Fig. S1A). For *O*s*WRKY74* overexpression, the full-length cDNA of *O*s*WRKY74* was amplified using two primers, 5′-CGC*GGATCC*ATGGAGAGCATGGAGGGCAATGG-3′, (*Bam*H I site underlined) and 5′-CGG*GGTACC*TCATGC GAAGAAGCTGGTGAT-3′, (*Kpn* I site underlined), by RT-PCR with Pyrobest DNA Polymerase (TaKaRa), directionally cloned into the *Kpn*I-*Bam*HI sites of a pUN1301 under the control of the ubiquitin promoter (Supplementary Fig. S1B). These constructs were electroporated into *Agrobacterium tumefaciens* EHA105 and transformed into rice (Zhonghua 10). Generation of transgenic rice plants was performed as described by Ge *et al.* (2004). T2 and T3 seeds were used for subsequent experiments.

### DNA gel blot analysis

Genomic DNA isolated from 2-week-old rice seedlings was digested with *Hin*d Ш, fractioned electrophoretically on 0.8% (w/v) agarose gel, and blotted onto a nylon membrane (Amersham Pharmacia Biotech). α-^32^P-ATP- and CTP-labelled *GUS* amplified from pUN1301 was used as a probe for hybridization. The membrane was exposed to x-ray film (Eastman-Kodak) at −70°C for 3–7 d.

### Determination of P and Fe

The dry root and shoot samples were separated and digested with concentrated nitric acid and hydrogen peroxide, and total P and Fe were determined by using inductively coupled plasma mass spectrometry following the protocols described by Song *et al.* (2011).

### Qualitative analysis of root-associated APase activity

Root APase staining was analysed according to Bozzo *et al.* (2006). The roots were excised from 14-d-old Pi-supplied and Pi-deprived seedlings and incubated with a 5-bromo-4-chloro-3-indolyl-phosphate (BCIP) agar overlay solution containing 50mM sodium acetate (pH 5.5) with 10mM MgCl_2_, 0.6% agar and 0.08% BCIP at room temperature for 20min. The blue colour on the root surface, formed by hydrolysis of BCIP, was photographed using a PENTAX k-7 camera (Pentax Corporation, Tokyo, Japan).

### Protein extraction and APase activity assay

Protein was isolated with ice-cold extraction buffer (100mM potassium acetate, pH 5.5, 20mM CaCl_2_, 2mM EDTA, 1mM dithiothreitol, 0.1mM phenylmethylsulfonyl ﬂuoride and 1.5% (w/v) polyvinylpolypyrrolidone) from 0.5mg of roots of 14-d-old seedlings. The protein content was determined using the method of Bradford (1976), with BSA as an internal standard. APase activity was analysed as described by Tomscha *et al.* (2004). Acid phosphatase activity was assayed by adding 1 μg of protein to 620 μl of reaction buffer (50mM NaAc pH 5.5 and 10mM MgCl_2_), and 10 ul of p-nitrophenol phosphate (10mg ml^-1^ pNPP; Sigma). After incubation at 37°C for 10min, the reaction was stopped by 1.2ml of 1M NaOH, and then absorbance was measured at 412nm wavelength. Phosphatase activity was expressed as ng of pNPP accumulated μg^–1^ soluble protein min^–1^. These experiments were replicated three times.

### Statistical analyses

For statistical analyses, the SPSS Statistics Base software package (version 16) was used. Significant differences were evaluated using one-way ANOVA and Duncan’s test at *P*≤0.05.

## Results

### Structural features, phylogenetic tree, and subcellular localization of OsWRKY74

**Fig. 1.**
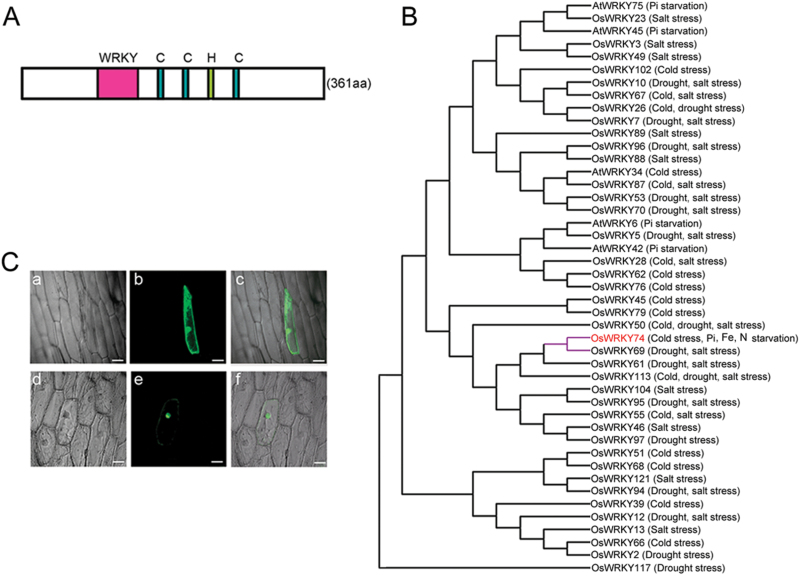
Structure, localization, and phylogenetic tree of OsWRKY74. (A) Scheme showing the structure of OsWRKY74 protein. aa, amino acids. (B) Phylogenetic tree of WRKY proteins. The tree was constructed with the MEGA 6.0 tree program with amino acid sequences of OsWRKY74 and other members of the WRKY family isolated from Arabidopsis and rice. The full-length amino acid sequences were downloaded from the institute for Genomic Research (http://www.tigr.org) and the National Center for Biotechnology information (http://www.ncbi.nlm.nih.gov). (C) Localization of OsWRKY74-GFP protein. Individual panels show GFP alone (b) or OsWRKY74-GFP (e) in onion epidermal cells, corresponding bright-field images (a and d), and merged images (c and f) of a and b and of d and e, respectively. GFP and OsWRKY74-GFP fusion was driven by the control of the CaMV 35S promoter. Onion epidermal peels were bombarded with DNA-coated gold particles, and GFP expression was visualized 24h later. Bars, 50 µm.

*OsWRKY74* (LOC_Os09g16510) was identified from a low Pi-responsive rice microarray. A Blastp search revealed that OsWRKY74 protein had a highly conserved WRKYGQK motif and a characteristic C2-HC zinc finger motif (Fig. 1A). Therefore, this gene belongs to group III of the WRKY TFs. A search of PROSITE (http://www.expasy.org/prosite) revealed that the OsWRKY74 protein contained 13 potential protein kinase phosphorylation sites and an Ala-rich domain in its C-terminal (Supplementary Fig. S2).

Most WRKY proteins studied so far have been implicated in regulating biotic stress responses. However, recent studies revealed that WRKY TFs are closely associated with abiotic stresses such as nutrient deficiency, cold, salt and drought stresses (Rushton *et al.*, 2010, Chen *et al.*, 2012, Yokotani *et al.*, 2013). To better understand the role of OsWRKY74 under conditions of various abiotic stresses (cold, drought, salinity or Pi starvation), we analysed WRKY proteins involved in abiotic stress from rice based on the report by Ramamoorthy *et al.* (2008), and WRKY proteins have been characterized for their functions in tolerance to cold stress and Pi starvation in rice and Arabidopsis. Subsequently, a phylogenetic tree was constructed using the MEGA 6.0 program. Phylogenetic analysis revealed that OsWRKY74 was not grouped with AtWRKY42, AtWRKY45, AtWRKY6 and AtWRKY75 WRKY proteins involved in Pi homeostasis; rather, it formed a separate branch with OsWRKY69 belonging to group III of the WRKY proteins with unknown function (Fig. 1B), although OsWRKY69 was induced by drought and salt stress (Ramamoorthy *et al.*, 2008).

To determine its subcellular localization, *O*s*WRKY74* was fused in frame to a 5′ terminus of the *GFP* reporter gene under the control of the cauliflower mosaic virus 35S (CaMV 35S) promoter. The recombinant constructs of the *O*s*WRKY74-GFP* fusion gene and *GFP* alone were introduced into onion (*Allium cepa*) epidermal cells by the particle bombardment. The OsWRKY74-GFP fusion protein accumulated mainly in the nucleus, whereas GFP alone was present throughout the whole cell (Fig. 1C), suggesting that OsWRKY74 is a nucleus-localized protein. This result is also consistent with the predicted function of OsWRKY74 as a TF.

### Expression patterns of *OsWRKY74*

**Fig. 2.**
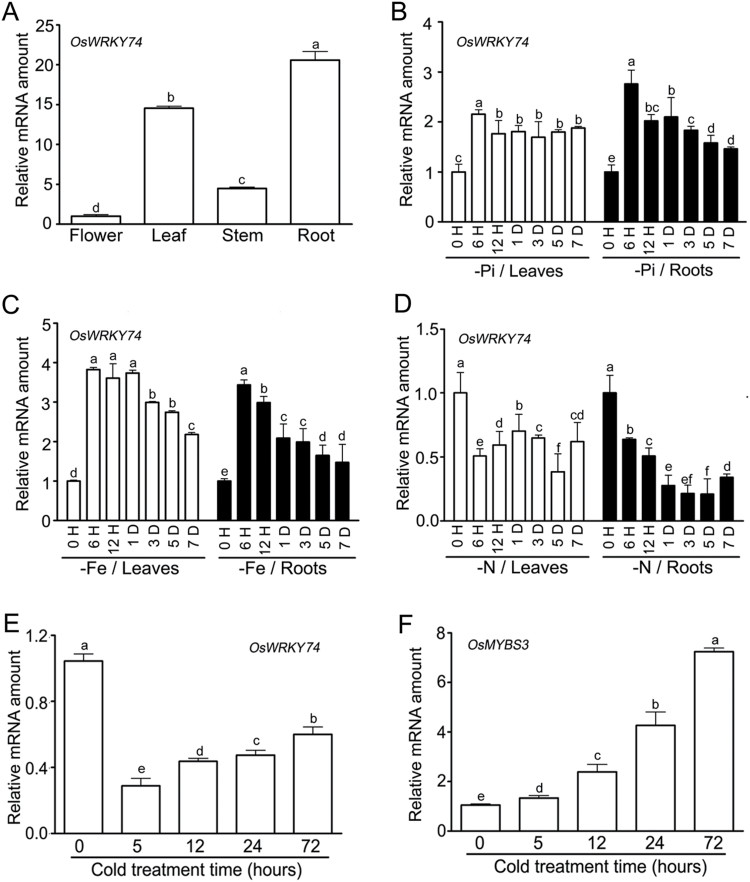
Expression patterns of *OsWRKY74* in different organs, and effect of the deprivation of Pi, N and Fe on the expression of *OsWRKY74*. (A) *OsWRKY74* expression in different tissues. (B) Time-course of *OsWRKY74* expression in response to Pi deprivation. Response of *OsWRKY74* to deprivation of (C) Fe and (D) N. (E) Time-course of *OsWRKY74* expression in response to cold stress. (F) Time-course of *OsMYS3* expression in response to cold stress. *Actin* was used as an internal control. Expression was normalized to that of *Actin*. Data are means ±SD (*n*=3). Means with different letters are significantly different (one-way ANOVA, Duncan, *P*≤0.05).

Quantitative real-time RT-PCR analysis showed that *OsWRKY74* was expressed in all organs examined, with the highest expression in roots and lowest in flowers (Fig. 2A). The expression patterns of *OsWRKY74* under Pi-sufficient and Pi-deficient conditions were evaluated by real-time RT-PCR using RNA samples extracted from roots and leaves. As shown in Fig. 2B, Pi starvation-induced expression of *OsWRKY74* was observed in roots and leaves up to 7 d. The increases in *OsWRKY74* transcripts peaked at 6h of Pi starvation and displayed a gradually decline thereafter (Fig. 2B). To determine whether the up-regulation of *OsWRKY74* gene was specific to Pi-starvation, the responsiveness of *OsWRKY74* expression to deprivation of other mineral nutrients, including nitrogen (N), potassium (K), and iron (Fe), was also investigated. Similar to Pi deprivation, the expression of *OsWRKY74* was markedly enhanced by deprivation of Fe (Fig. 2C). In contrast, the expression of *OsWRKY74* was suppressed by deprivation of N (Fig. 2D). No significant difference in *OsWRKY74* mRNA by deprivation of K up to 7 d was observed (Supplementary Fig. S3).

The transcript of *OsWRKY74* was down-regulated after 5h of cold treatment and the down-regulation lasted up to 72h of cold treatment (Fig. 2E). To further validate this experiment, we used *MYBS3* (Fig. 2F), a gene encoding MYB protein from rice, as a positive control. *MYBS3* is induced by cold stress (Su *et al.*, 2010). In contrast, no response of *OsWRKY74* was detected when treated with salt and dehydration stress for 5h (Supplementary Fig. S4). Taken together, these results suggest that *OsWRKY74* is induced by deficiency of Pi and Fe, while it is suppressed by N deficiency and cold stress.

### Molecular characterization of *OsWRKY74*-overexpressed and RNAi knockdown transgenic lines

**Fig. 3.**
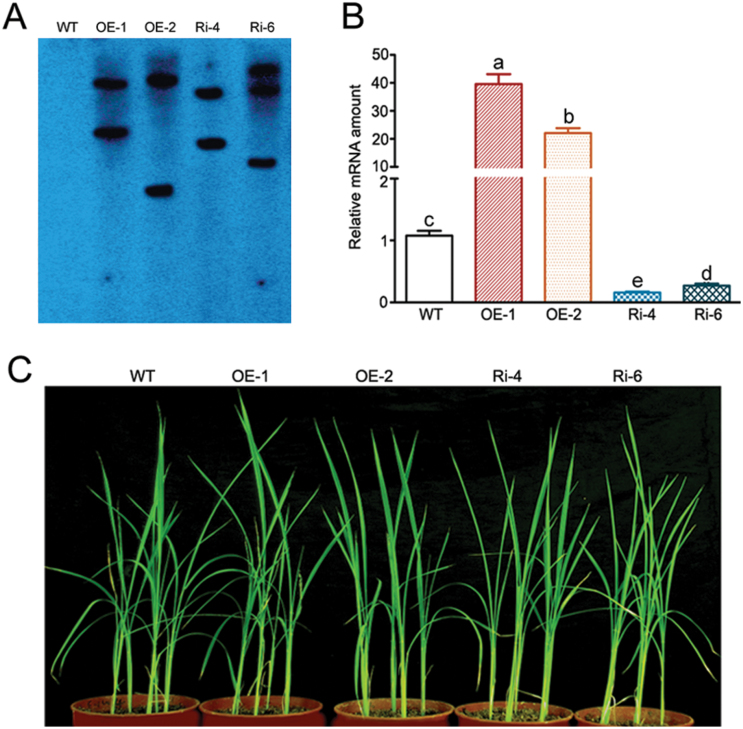
Molecular characterization and phenotypes of *OsWRKY74* transgenic plants. (A) Southern-blot assay for rice transgenic plants. Genomic DNA isolated from WT and transformed plants digested with *Hin*d Ш. The blot was hybridized with the ORF of the *GUS* gene labelled with α-^32^P-dCTP and α-^32^P-dATP as described in Materials and Methods. (B) Expression of independent transgenic rice by real-time PCR analysis. Expression was normalized to that of *Actin*. The transcript level from the WT was set to 1. Data are means ±SD (*n*=3). Means with different letters are significantly different (one-way ANOVA, Duncan, *P*≤0.05). (C) The phenotypes of the WT and T3 transgenic plants after growing in soil for 30 d.

To investigate the function of *OsWRKY74* in planta, we overexpressed and suppressed *OsWRKY74* in rice under the control of a ubiquitin promoter of maize. Transgenic rice lines of *OsWRKY74* were confirmed by hygromycin selection and Southern blotting. Southern blotting was performed using the DNA digested with *Hind* III and the GUS gene as a probe. Two overexpressed lines and two RNA interference (RNAi) lines were randomly selected, and different hybridized patterns to the *GUS* probe were observed. In contrast, no signals were detected in WT rice plants under the same conditions (Fig. 3A). Therefore, the two overexpressed transgenic lines and two RNAi transgenic lines are likely to be independent. Furthermore, real-time PCR analysis showed that expression of *OsWRKY74* was markedly increased in the two independent overexpressing lines, while its expression was suppressed in the two RNAi transgenic lines (Fig. 3B).

To examine the phenotypes of transgenic lines, T3 transgenic lines and the WT were grown in a greenhouse under identical conditions. Seedlings of both overexpressing and knockdown lines showed comparable phenotypes to WT plants under non-stressed, control conditions (Fig. 3C), suggesting that alteration of *OsWRKY74* expression has no impacts on their phenotypes under normal, non-stressed conditions.

### Response of rice lines expressing *OsWRKY74* to Pi starvation

**Fig. 4.**
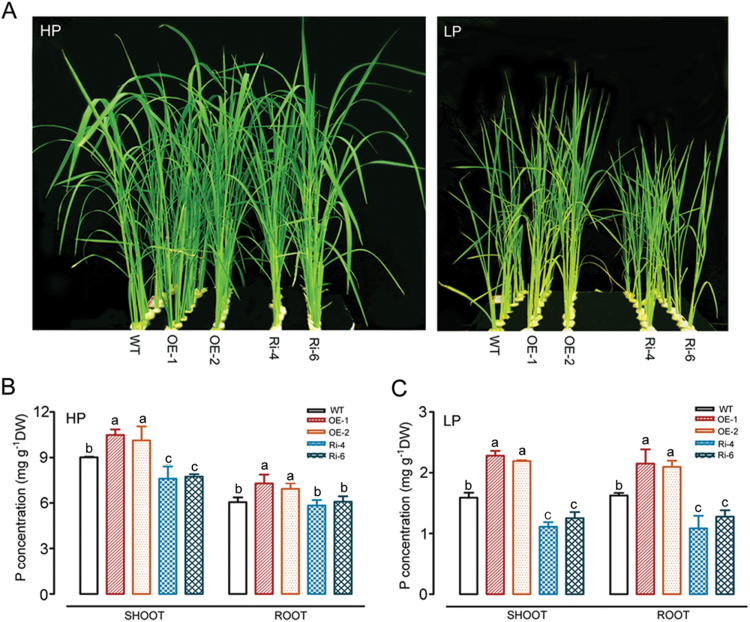
Effect of *OsWRKY74* expression on tolerance to Pi deficiency in hydroponics. (A) The phenotypes of WT, *OsWRKY74*-overexpressing and *OsWRKY74* RNAi plants grown in the greenhouse for 30 d under HP or LP conditions. Plants were pregerminated in water for 7 d and grown hydroponically for 30 d in medium containing 0.323 or 0.0161mM Pi. P concentration in roots and shoots of WT, *OsWRKY74*-overexpressing and *OsWRKY74* RNAi knockdown plants grown in the greenhouse for 30 d under (B) HP and (C) LP conditions. Data are means of three replicates with errors bars indicating SD. Means with different letters are significantly different (one-way ANOVA, Duncan, *P*≤0.05). DW, dry weight.

To functionally characterize the role of *OsWRKY74* in response and adaptation to Pi starvation, 1-week-old plants of T3 transgenic lines and the WT were exposed to a hydroponic solution containing a high level of Pi (HP; 0.323mM Pi) and a low level of Pi (LP; 0.016mM Pi) for 30 d. In the hydroponic culture solution with a high-Pi level, no significant differences in plant height, root and shoot biomass were observed between WT and transgenic plants (Fig. 4A and Table 1). With a low-Pi level, however, the *OsWRKY74*-overexpressing lines (OE-1 and OE-2) exhibited better growth than WT plants, as evidenced by the greater plant height, P concentration, root and shoot biomass of the transgenic plants compared to the WT plants (Fig. 4 and Table 1). In contrast to the overexpressing lines, growth of the RNAi transgenic lines (Ri-4 and Ri-6) was less than that of the WT plants, as evidenced by the shorter plant height, and lower P concentration, shoot and root biomass of the knockdown transgenic plants compared to the WT plants when grown in LP medium (Fig. 4 and Table 1). These results suggest that interference of *OsWRKY74* renders rice seedlings more sensitive to Pi deficiency.

**Table 1.** Plant height, dry shoot biomass and dry root biomass of wild-type and transgenic plants from solution culture experiments

Genotype	Shoot biomass (g DW)	Root biomass (g DW)	Plant height (cm)
HP (0.323mM Pi)			
Wild type	1.816±0.037^a^	0.276±0.002^a^	56.2±0.56^a^
OE-1	1.881±0.061^a^	0.282±0.031^a^	57.4±0.41^a^
OE-2	1.795±0.054^a^	0.280±+0.024^a^	56.8±1.60^a^
Ri-4	1.885±0.043^a^	0.278±0.008^a^	55.5±1.00^a^
Ri-6	1.945±0.054^a^	0.272±0.015^a^	55.9±0.67^a^
LP (0.0161mM Pi)			
Wild type	0.798±0.003^b^	0.189±0.005^b^	41.16±0.15^b^
OE-1	1.035±0.016^a^	0.238±0.006^a^	47.48±2.08^a^
OE-2	0.931±0.001^a^	0.219±0.019^a^	47.06±2.15^a^
Ri-4	0.527±0.014^c^	0.124±0.004^c^	34.02±1.29^c^
Ri-6	0.579±0.022^c^	0.128±0.013^c^	37.97±2.17^c^

Plants were pre-germinated in water for 7 d and grown hydroponically for 30 d in medium containing HP or LP, and then plants were sampled for the measurements. The values are mean ± SD of three independent experiments, with 10 seedlings being used in each experiment. Means with different letters are significantly different (one-way ANOVA, Duncan, *P* ≤ 0.05). DW, dry weight.

**Fig. 5.**
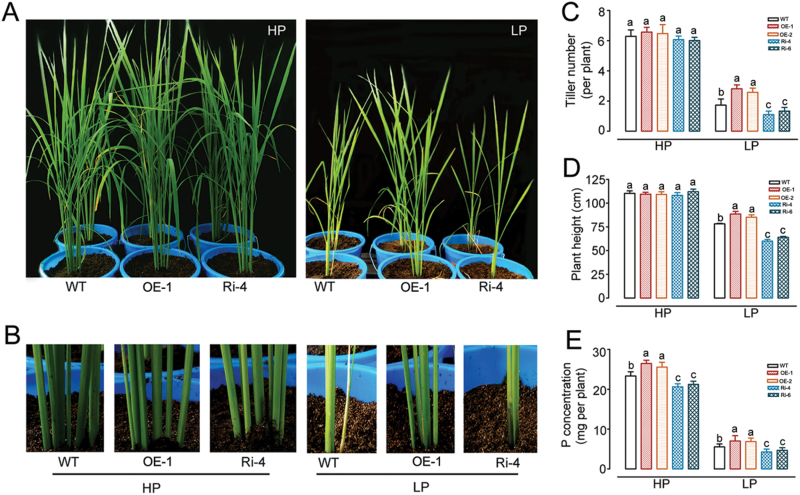
Effect of *OsWRKY74* expression on tolerance to Pi deficiency when grown in soil. (A) Growth of WT and transgenic rice plants at 4 months in Pi-sufficient or Pi-deficient soil (plant height was studied after harvest). (B) Comparison of tiller numbers between WT and transgenic rice plants. Wild-type, *OsWRKY74*-overexpressing and *OsWRKY74* RNAi lines are shown after 4 months of growth under the Pi-sufficient or Pi-deﬁcient conditions. (C) Quantitative analysis of the tiller number as shown in panel B. (D) Plant height and (E) P concentration of WT and transgenic rice plants at 4 months in Pi-sufficient or Pi-deficient soil. Data are means ±SD of three independent experiments, with 8 seedlings in each experiment. Means with different letters are significantly different (one-way ANOVA, Duncan, *P*≤0.05).

To further confirm the tolerance of *OsWRKY74* in response to Pi deficiency, the transgenic lines and WT plants were grown in soil with two levels of Pi supply (high and low Pi soils contained 60 and 15mg Pi kg^-1^ soil, respectively) for the entire growth period (~150 d). In the low-Pi soils, plant height, tiller number, shoot and root biomass, grain weight, and P concentration of the *OsWRKY74*-overexpressing plants were about 13%, 45%, 37%, 35%, 33% and 24% higher than those in WT plants, respectively (Fig. 5 and Table 2). In contrast, the RNAi knockdown lines exhibited greater growth inhibition than WT plants. For instance, the *OsWRKY74* RNAi transgenic lines had fewer tillers (1.7 per plant on average) than WT plants (1.2 per plant on average) in the whole growth period. In addition, the grain weight, P concentration and shoot biomass were also lower in the RNAi lines than in the WT (Fig. 5 and Table 2). In the high-Pi soils, however, no signiﬁcant differences in tiller number, shoot biomass, grain weight and plant height were observed between WT and transgenic plants (Fig. 5 and Table 2). Taken together, these results suggest that the expression level of *OsWRKY74* in rice plants is positively correlated with tolerance to low Pi stress.

**Table 2.** Dry shoot biomass, dry root biomass and grain weight of each plant at harvest stage of wild-type and transgenic plants from pot soil experiments

Genotype	Shoot biomass (g DW)	Root biomass (g DW)	Grain weight (g per plant)
60mg Pi soil^-1^			
Wild type	45.984±1.671^a^	7.586±0.846^a^	9.804±1.423^a^
OE-1	43.211±1.114^a^	8.041±0.642^a^	10.124±0.824^a^
OE-2	44.463±0.973^a^	7.948±+0.586^a^	9.648±1.112^a^
Ri-4	43.078±1.034^a^	7.146±0.467^a^	9.402±1.687^a^
Ri-6	44.116±1.042^a^	7.018±0.4365^a^	10.002±1.058^a^
15mg Pi soil^-1^			
Wild type	11.879±1.001^b^	2.962±0.817^b^	3.024±0.987^b^
OE-1	17.164±1.224^a^	4.108±0.516^a^	4.214±0.632^a^
OE-2	16.345±0.668^a^	4.027±0.535^a^	4.026±0.548^a^
Ri-4	9.467±0.985^c^	1.987±0.644^c^	2.504±0.669^c^
Ri-6	9.942±1.045^c^	2.168±0.713^c^	2.538±0.776^c^

Two Pi fertilizer levels were employed at 60mg Pi/kg and 15mg Pi/kg. Each pot contained 15kg soil and individual plants were transplanted into each pot (n = 8). The values are mean ± SD of three-pot experiments, with 8 seedlings being used in each-pot experiment. Means with different letters are significantly different (one-way ANOVA, Duncan, *P* ≤ 0.05). DW, dry weight

### *OsWRKY74* is involved in regulating acid phosphatase activities

**Fig. 6.**
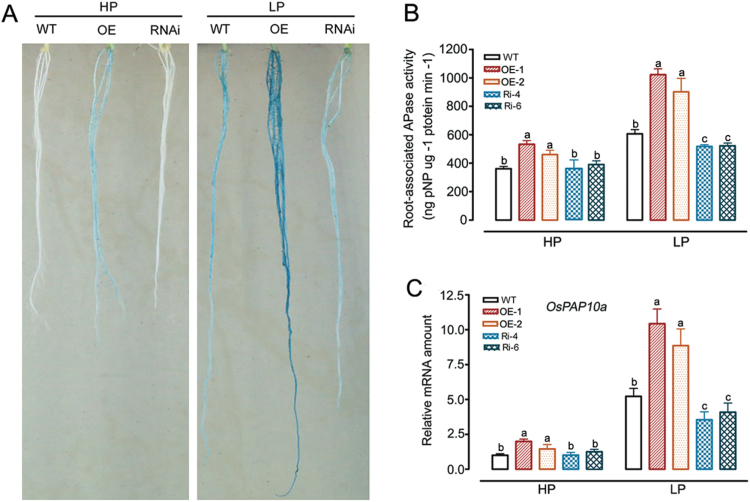
Analysis of acid phosphatase activities in WT and *OsWRKY74* transgenic plants. (A) The phenotypes of WT, *OsWRKY74*-overexpressing and *OsWRKY74* RNAi plants on BCIP. Germinated seeds were cultured in HP or LP conditions for 14 d. Roots of the plants were then sampled for BCIP staining. (B) Root-associated APase activity. (C) Relative qRT-PCR expression analysis of gene encoding rice acid phosphatase (*OsPAP10a*) in roots of WT and *OsWRKY74* transgenic plants under HP or LP conditions. Expression was normalized to that of *Actin*. Data are means ±SD (*n*=3). Means with different letters are significantly different (one-way ANOVA, Duncan, *P*≤0.05).

An increase in activity of root acid phosphatases (APase) is a common phenomenon in the response of plants to Pi starvation. To test whether APase activity is involved in the OsWRKY74-mediated responses to Pi deficiency, APase activities in roots of WT and transgenic plants were visualized in plants grown in both HP and LP media. The APase activities in *OsWRKY74*-overexpressing roots were much higher than in the WT and RNAi plants under both HP and LP conditions, as indicated by the stronger blue staining of *OsWRKY74*-overexpressing roots than that of WT and RNAi plants (Fig. 6A). Higher APase activities in the *OsWRKY74*-overexpressing roots than in WT and RNAi roots in HP and LP media were also obtained by determining hydrolytic activity in roots on substrate pNPP (Fig. 6B). Consistently, the gene coding for purple acid phosphatase10a (*OsPAP10a*), was also up-regulated in O*sWRKY74*-overexpressing roots in HP and LP solutions (Fig. 6C). Together, these results indicate that OsWRKY74 can stimulate acid phosphatases to facilitate Pi acquisition.

### *OsWRKY74*-mediated Pi acquisition may depend partly on changes in root system architecture

**Fig. 7.**
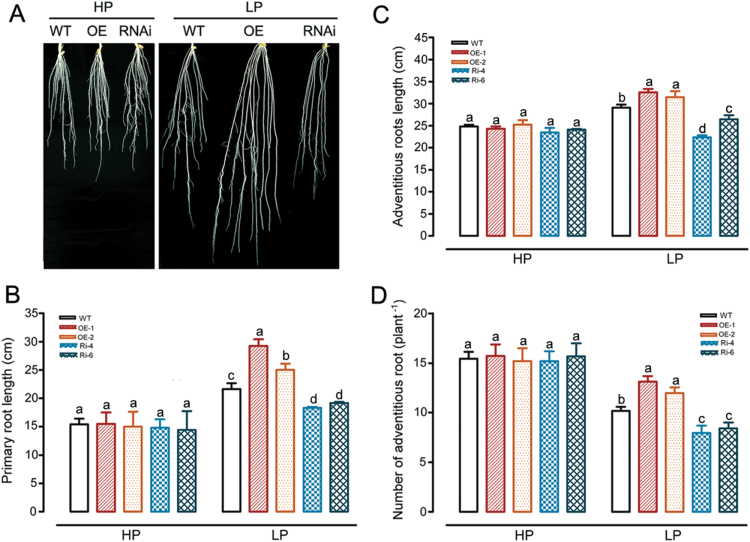
Effect of Pi availability in the medium on root system architecture in WT and transgenic rice. (A) Root system architecture of WT, *OsWRKY74*-overexpressing and *OsWRKY74* RNAi plants grown in Pi-sufficient (HP, 0.323mM; left) and Pi-deficient (LP, 0.016mM; right) media for 14 d. Quantitative analysis of the length of primary roots (B), the length of three longest adventitious roots (C), and the number of adventitious roots, (D) of WT and *OsWRKY74*-overexpressing and *OsWRKY74* RNAi rice seedlings after grown in the HP or LP medium for 14 d. Error bars indicate SD. Means with different letters are significantly different (one-way ANOVA, Duncan, *P*≤0.05).

Root system architecture (RSA) is an important root trait that is sensitive to Pi status in growth medium (López-Bucio *et al.*, 2003; Jain *et al.*, 2007). To test whether the greater growth of transgenic rice plants grown in LP medium is related to changes in the root system architecture, 1-week-old WT and transgenic plants with overexpressing and RNAi lines of *OsWRKY74* grown in hydroponic solution containing high and low Pi for 14 d were used to compare the number of adventitious roots, primary root length and total length of the three longest adventitious roots. No significant differences in root system architecture were observed between WT and the transgenic lines when grown in HP medium (Fig. 7). However, the elongation of primary and adventitious roots was significantly enhanced in *OsWRKY74*-overexpressing plants compared with WT plants under LP conditions (Fig. 7B, C). Moreover, the number of adventitious roots in the *OsWRKY74*-overexpressing plants was much greater than that in WT plants in LP medium (Fig. 7D). These results suggest that the improved growth of the *OsWRKY74*-overexpressing lines in Pi-deficient conditions may be attributable, at least partially, to the larger root system.

### *OsWRKY74* regulates the expression of phosphate-responsive genes

**Fig. 8.**
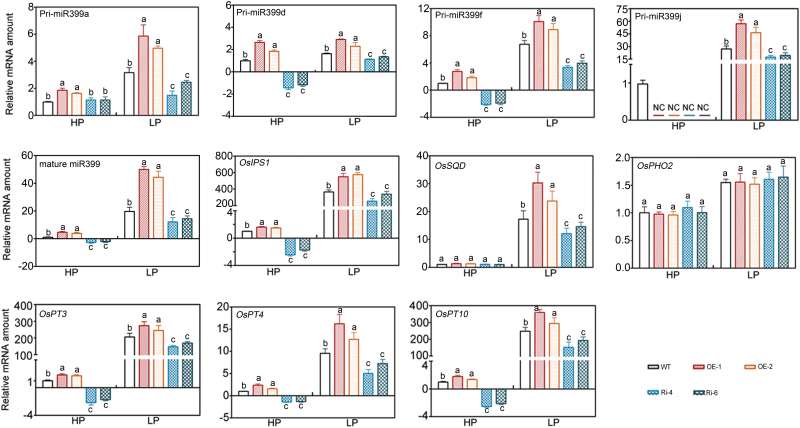
Expression of Pi starvation-induced genes in WT and *OsWRKY74* transgenic plants. Total RNA samples were extracted from roots of seedling grown in normal nutrient solution for 7 d, followed by treatment with HP or LP medium for 14 d. Expression was normalized to that of *Actin*. Data are means ±SD (*n*=3). Means with different letters are significantly different (one-way ANOVA, Duncan, *P*≤0.05).

To further elucidate the mechanisms underlying the regulation of Pi starvation response by OsWRKY74, the expression of several Pi-starvation inducible (PSI) genes was monitored by real-time PCR. The Pi-starvation inducible genes, including *Oryza sativa* UDP-sulfoquinovose synthase (*OsSQD*), *OsIPS1*, *OsmiR399a*, *OsmiR399d*, *OsmiR399f* and *OsmiR399j*, were markedly induced in both WT and *OsWRKY74*-overexpressing transgenic plants when grown in LP medium (Fig. 8). This observation is in line with results reported in previous studies (Essigmann *et al.*, 1998; Yu, *et al.*, 2002; Hou *et al.*, 2005; Zhou *et al.*, 2008). However, the expression of *OsSQD*, *OsIPS1*, *OsmiR399a*, *OsmiR399d*, *OsmiR399f* and *OsmiR399j* in both roots and shoots of *OsWRKY74*-overexpressing plants was significantly higher than in WT plants in LP medium (Fig. 8; Supplementary Fig. S5). In contrast to *OsWRKY74*-overexpressing transgenic plants, the expression of *OsSQD*, *OsIPS1*, *OsmiR399a*, *OsmiR399d*, *OsmiR399f* and *OsmiR399j* in both roots and shoots of RNAi *OsWRKY74* was significantly lower than that in WT plants in both HP and LP media (Fig. 8; Supplementary Fig. S5). These results suggest that the OsWRKY74 protein may modulate Pi starvation by partially altering expression of downstream Pi-starvation inducible genes.

Thirteen putative genes encoding high-affinity Pi transporters have been identified in rice (Paszkowski *et al.*, 2002). We evaluated the effect of *OsWRKY74* on expression of these genes. We failed to detect the expression of *OsPT11* and *OsPT13* in both WT and transgenic plants grown in both HP and LP media. These results are consistent with reports demonstrating that *OsPT11* and *OsPT13* are exclusively induced in roots by inoculation with arbuscular mycorrhiza fungi (Paszkowski *et al.*, 2002; Glassop *et al.*, 2005). Disruption of *OsWRKY74* expression in transgenic rice plants altered the expression of *OsPT3*, *OsPT4* and *OsPT10* under both HP and LP conditions (Fig. 8; Supplementary Fig. S5), while expression of the remaining *PHT* genes in WT plants was comparable to that in the transgenic rice plants under both HP and LP conditions (data not shown). Thus, *OsWRKY74* may regulate Pi acquisition by targeting *OsPT3*, *OsPT4* and *OsPT10* transporters under LP conditions.

## Discussion

### *OsWRKY74* encodes a WRKY TF responsive to deficiencies of Pi, Fe, N and cold stress

The WRKY protein family is a plant-specific transcription factor, and the emerging evidence indicates the important roles played by WRKY proteins in response to nutrient deficiency (Chen *et al.*, 2012). In the rice genome, the WRKY TF family comprises 109 members (Ross *et al.*, 2007), but the roles of WRKY TFs involved in the maintenance of Pi homeostasis in rice are poorly understood. Our results showed that the expression of *OsWRKY74* was rapidly up-regulated by Pi and Fe deprivation, and repressed by N deprivation (Fig. 2), implying its involvement in regulating multiple nutrient starvation responses in rice. This expression pattern differs from that of other Pi-responsive WRKY TFs previously reported. For instance, *AtWRKY6* expression is activated in response to Pi deficiency, boron and arsenate starvation (Robatzek and Somssich, 2002; Chen *et al.*, 2009; Kasajima *et al.*, 2010; Castrillo *et al.*, 2013). *AtWRKY75* is strongly induced upon Pi starvation and pathogen infection (Dong *et al.*, 2003; Devaiah *et al.*, 2007a). *AtWRKY45* is induced during Pi starvation, mainly in the roots (Wang *et al.*, 2014). The transcript of *AtWRKY42* was suppressed under Pi-deficient conditions (Su *et al.*, 2015). Thus, this is the ﬁrst report, to our knowledge, showing that *OsWRKY74* is involved in the regulation of multiple nutrient starvations in rice.

**Fig. 9.**
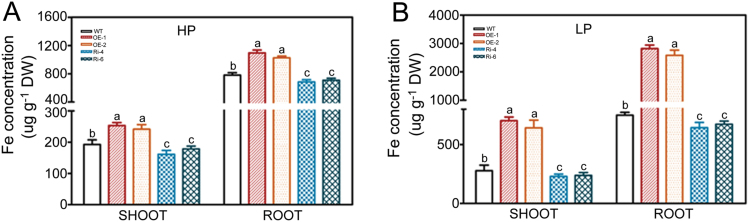
Fe concentration in WT and *OsWRKY74* transgenic plants. (A, B) Fe concentration in roots and shoots of WT, *OsWRKY74*-overexpressing and *OsWRKY74* RNAi knockdown plants grown in the greenhouse for 30 d under HP or LP conditions. Data are means of three replicates with errors bars indicating SD. DW, dry weight. Means with different letters are significantly different (one-way ANOVA, Duncan, *P*≤0.05).

An increase in metallic element acquisition is another adaptive response to Pi starvation in plants (Wasaki *et al.*, 2003; Misson *et al.*, 2005). Pi deprivation leads to an increase in Fe contents and activation of Fe-responsive genes (Hirsch *et al.*, 2006; Zheng *et al.*, 2009; Bournier *et al.*, 2013). In our study, we found that overexpression of *OsWRKY74* enhanced the accumulation of Fe in both roots and shoots regardless of Pi status in the growth medium (Fig. 9), implying that *OsWRKY74* may also play a regulatory role in Fe homeostasis in addition to control of P acquisition. *MiR399* has been established as an important component in the Pi starvation signalling network (Fujii *et al.*, 2005). Recent studies reported that *miR399* is significantly induced by Fe starvation in both roots and shoots and the concentration of Fe is increased in the *OsmiR399*-overexpressing plants (Hu *et al.*, 2015). Our results revealed that *OsmiR399* genes were up-regulated in *OsWRKY74*-overexpressing plants (Fig. 8; Supplementary Fig. S5). Therefore, *OsWRKY74* may play an important role in crosstalk between the Fe and Pi signalling cascades, which merits further study.

**Fig. 10.**
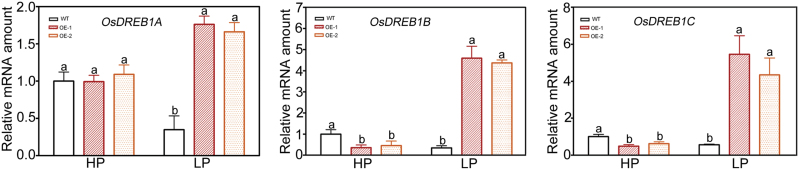
Effect of Pi deficiency on the expression of *DREB* genes in WT and transgenic plants. Total RNA samples were extracted from plants grown in normal nutrient solution for 7 d, followed by treatment with HP or LP medium for 14 d. Expression was normalized to that of *Actin*. Data are means ±SD (*n*=3). Means with different letters are significantly different (one-way ANOVA, Duncan, *P*≤0.05).

Interestingly, the expression of *OsWRKY74* was repressed upon exposure to cold stress (Fig. 2E). Two rice *WRKY* genes, *OsWRKY45* and *OsWRKY76*, have been functionally characterized for their roles in cold stress (Tao *et al.*, 2011; Yokotani *et al.*, 2013). The OsWRKY74 protein cannot be grouped with the OsWRKY45 and OsWRKY76 proteins; it was located in a separate branch (Fig. 1B). It has been proposed that Pi starvation and cold stress might share some common regulatory cascades, and that P may participate in the acclimatization to cold stress, as some cold-responsive genes are also regulated by Pi deficiency (Hammond *et al.*, 2003). In Arabidopsis and rice, the DREB1/CBF-dependent pathway is a central component of cold response, and overexpression of some CBFs results in enhancement of tolerance to freezing, salt and drought stress by activating associated target genes (Dubouzet *et al.*, 2003; Ito *et al.*, 2006). In the present study, the expression levels of *OsDREB1A*, *OsDREB1B* and *OsDREB1C* were significantly up-regulated in the *OsWRKY74*-overexpressing plants compared to the WT plants under LP conditions (Fig. 10). Therefore, *OsWRKY74* may play an important role in linking cold stress to Pi starvation signal transduction pathways.

### Overexpression of *OsWRKY74* confers tolerance to low-Pi stress in rice

Improvement of tolerance to low Pi stress by expressing Pi deficiency-induced TFs and/or protein kinase genes such as *PSTOL1*, *OsPTF1* and *PHR* has been reported in the literature (Yi et al, 2005; Nilsson et al, 2007; Gamuyao *et al.*, 2012). In this study, *OsWRKY74* overexpressors exhibited greater tolerance to low Pi stress in both the hydroponic and pot experiments, as indicated by an increased shoot, root biomass, grain weight and tiller number (Tables 1, 2; Figs 4, 5). The number of rice tillers is an important indicator for Pi nutrient status in plants, and is positively correlated with the tolerance to low-Pi stress (IRRI, 1996). In addition, rice genotypes with high Pi use efficiency often exhibit a better grain yield under conditions of low P availability in soils. In the *OsWRKY74*-overexpressing plants, an increase in grain yield was found under low-Pi conditions (Table 2). These results support the notion that *OsWRKY74* is a positive regulator of Pi starvation responses. Furthermore, the enhanced tolerance of *OsWRKY74*-overexpressing plants to low-Pi stress coincides with the up-regulation of low-Pi-responsive genes, including *OsPAP10a* and *OsSQD* (Figs 6C, 8). *OsPAP10a* encodes an acid phosphatase that is activated by Pi starvation in rice (Zhou *et al.*, 2008). *OsSQD* is involved in sulfolipid biosynthesis (Yu *et al.*, 2002; Zhou *et al.*, 2008). Enhanced production of acid phosphatases and activation of scavenging systems are adaptive mechanisms to maximize Pi availability for plants under low Pi conditions (Raghothama, 1999; Abel *et al.*, 2002). Thus, the enhanced tolerance of *OsWRKY74* transgenic plants to low Pi stress may depend in part on changes in the expression of these genes.

In addition, overexpression of *OsWRKY74* in rice plants conferred higher Pi content than WT plants under Pi-deficient conditions (Figs 4B, C, 5E). Pi transporters are directly responsible for Pi acquisition and transport in plants (Harrison *et al.*, 2002; Misson *et al.*, 2004). Plants generally up-regulate the expression of Pi transporters to enhance Pi uptake and transport efficiency to cope with low Pi supply in soils (Liu *et al.*, 1998; Karthikeyan *et al.*, 2002). In this study, several Pi transporters, such as *OsPT3*, *OsPT4* and *OsPT10*, may account for the Pi uptake in roots of *OsWRKY74*-overexpressing plants under Pi-deficient conditions, as evidenced by the greater up-regulation of these genes in *OsWRKY74*-overexpressing plants than in WT plants (Fig. 8). Therefore, *OsWRKY74* may regulate Pi acquisition by targeting PHT at the transcriptional level.

The *PHR*-*miR399*-*PHO2* pathway has been established to play a critical role in regulating Pi starvation response in Arabidopsis and rice (Rubio *et al.*, 2001; Fujii *et al.*, 2005; Bari *et al.*, 2006; Chiou *et al.*, 2006). Our results showed that the expression of *OsmiR399a*, *OsmiR399d, OsmiR399f* and *OsmiR399j* was enhanced in the *OsWRKY74*-overexpressing plants and repressed in the *OsWRKY74* RNAi transgenic plants under both HP and LP conditions (Fig. 8). These observations suggest that the positive regulation of *miR399* gene expression in response to Pi starvation is mediated at least in part by the TF *OsWRKY74*. As for *OsPHO2*, we did not find the *OsmiR399*-mediated transcript degradation as transcript abundance of *OsPHO2* was unchanged despite the change in *OsmiR399* levels (Fig. 8). Our data suggest that *OsWRKY74* may positively regulate *OsmiR399*, but *OsPHO2* may not be the target of *OsmiR399* in controlling Pi homeostasis in rice. It is also conceivable that its activity may be controlled by another level of post-transcriptional regulation that warrants further investigation.

Changes in the expression of genes involved in Pi signalling, high-afﬁnity Pi transport and mobilization highlight a global role of *OsWRKY74* in response to Pi deﬁciency (Fig. 8). In fact, it has been previously reported that the transcripts of an array of PSI genes involved in Pi sensing, translocation, transport and mobilization were reduced or induced by other TFs such as *ZAT6*, *MYB62*, and *AtERF070* (Devaiah et al., 2007b, 2009; Ramaiah *et al.*, 2014), but the precise mechanisms remain elusive. Thus, although up-regulation of PSI genes in *OsWRKY74*-overexpressing plants indicates that *OsWRKY74* may act as a positive regulator of their expression, more detailed studies are required to identify the targets of *OsWRKY74* and the genes controlled by *OsWRKY74* during Pi limitation.

Alterations in both primary and adventitious root elongation are a typical phenomenon in response to Pi deprivation in rice (Wissuwa, 2003; Yi *et al.*, 2005). In this study, the highest expression of *OsWRKY74* was observed in roots, but no significant difference in root system architecture was observed between WT and *OsWRKY74* transgenic plants in HP conditions. These results differ from those Pi-responsive TFs of *WRKY75*, *ZAT6*, *MYB62* and *OsMYB2P-1* in terms of their regulation of root system architecture (Devaiah et al., 2007a, b, 2009; Dai *et al.*, 2012). It has been reported that small changes in root system architecture can have marked effects on P uptake (Itoh and Barber, 1983; Wissuwa, 2003). Our results show that transgenic lines with an overexpression of *OsWRKY74* displayed significant increases in length of the primary and adventitious roots, as well as adventitious root number in LP medium (Fig. 7A–D). A similar increase in the primary and adventitious root length has been reported for *OsPTF1* and *OsMYB2P-1* overexpression lines (Yi *et al.*, 2005; Dai *et al.*, 2012). Thus, the larger root system of *OsWRKY74*-overexpressing plants grown in Pi-deficient conditions would allow the transgenic plants to exploit more soils and increase root surface area for Pi uptake, thus conferring them more efficient acquisition of Pi under Pi-deficient conditions.

## Conclusions

In summary, this study characterized a rice WRKY protein belonging to group III of the WRKY protein family that is localized at the nucleus in rice. The *OsWRKY74* protein acts as a positive regulator of Pi starvation responses, such that overexpression of *OsWRKY74* resulted in enhanced tolerance to low Pi stress, larger root system architecture under LP conditions, and improved expression of PSI genes. In addition, our results also showed that OsWRKY74 can function as a regulator of Fe starvation and cold stress. Further works on the role of OsWRKY74 crosstalk between P and Fe, and P and cold stress are under way in our laboratory.

## Supplementary data

Supplementary data is available at *JXB* online.

Figure S1. Plasmids construction for plant transformation.

Figure S2. Deduced amino acid sequence of OsWRKY74.

Figure S3. Response of *OsWRKY74* to -K in leaves and roots.

Figure S4. Expression of *OsWRKY74* under cold, NaCl and drought treatments.

Figure S5. Expression of Pi starvation-induced genes in wild-type (WT) and *OsWRKY74* transgenic plants.

Table S1. Primers used in semi-quantitative RT-PCR and real-time RT-PCR.

Table S2. Sequences of forward and reverse primers and 6-FAM 5′ end-labelled probes designed for the 3′ UTR of the rice Pi transporter genes and the rice polyubiquitin 1 (RubQ1) gene for quantitative RT-PCR.

Supplementary Data
