# Supplementary material for: OsWRKY74, a WRKY transcription factor, modulates tolerance to phosphate starvation in rice
Source: J Exp Bot. 2015 Dec 11;67(3):947–60. doi: 10.1093/jxb/erv515 (PMC4737085; doi:10.1093/jxb/erv515)
Supplement: Supplementary Data [file supp_67_3_947__index.html]

 OsWRKY74, a WRKY transcription factor, modulates tolerance to phosphate starvation in rice — OsWRKY74, a WRKY transcription factor, modulates tolerance to phosphate starvation in rice — Supplementary Data 

# *OsWRKY74*, a WRKY transcription factor, modulates tolerance to phosphate starvation in rice

## Supplementary Data

Data files

- Supplementary\_figures\_S1\_S5\_Tables\_S1\_S2.pdf - Supplementary Data
