# Supplementary material for: OsWRKY74, a WRKY transcription factor, modulates tolerance to phosphate starvation in rice
Source: J Exp Bot. 2015 Dec 11;67(3):947–60. doi: 10.1093/jxb/erv515 (PMC4737085; doi:10.1093/jxb/erv515)
Supplement: Supplementary Data [file supp_erv515_Supplementary_figures_S1_S5_Tables_S1_S2.pdf]

Supplementary Fig. S1

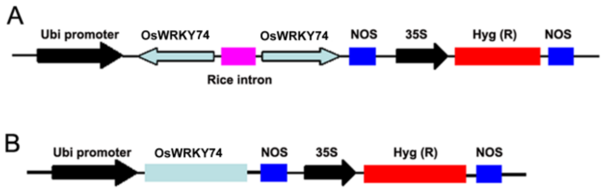

Supplementary Fig. S1. Plasmids construction for plant transformation. (A) schematic representation of the RNAi construct of *OsWRKY74* used for rice transformation. (B) schematic representation of the over-expressed construct of *OsWRKY74* used for rice transformation.

Supplementary Fig. S2

```
1     MESMEGNGGG RLVVTELSHI KELVRQLEGH LGGSGSPDLC
41    KHLASQIFSV TERSIGMIRS GHFDGHRKRS AAAVAAGDLD
81    SATPSPLSDV SDLPFKATKK RKTSTEKKRH QIRVSSTGGV
121   ENPPVDDGHS WRKYGQKEIL GAKHPRGYYR CTHRHSQGCM
161   ATKQVQRTDE DATVFDVIYH GEHTCVHKAV AAGAGKPETE
201   TDTNAAAESR LHDLSSGLTV KIEGLTAPPQ QQQGGGGWNA
241   MPPFCLSSPV SGLAPPDQHN PFSAPSTPEN RLAAAASSAA
281   SPATSDSMAA APFHQAAAGG GDEAWRDAEL QEVVSALVAA
321   TTTTATAQPA PATAMVDADL SALDAFEFDP GFTIDITSFF
361   A
```

Supplementary Fig. S2. Deduced amino acid sequence of OsWRKY74. Thirteen predicted Protein kinase phosphorylation site are highlighted in yellow on the sequence. Alanine-rich region is highlighted in green on the sequence. WRKY domain is highlighted with dot lines on the sequence. WRKYGQK and C_2HC motif are highlighted in blue and red, respectively.

Supplementary Fig. S3

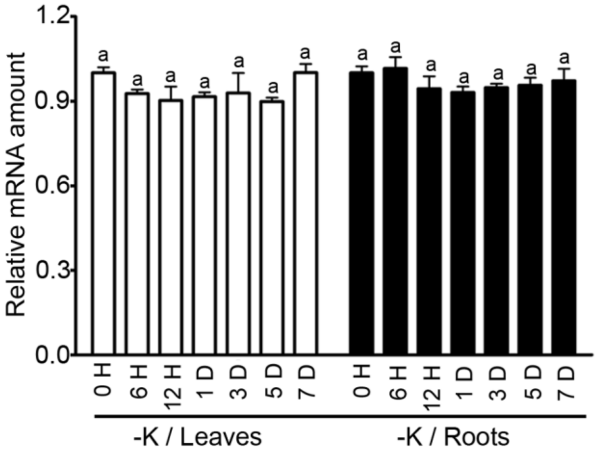

Supplementary Fig. S3. Response of *OsWRKY74* to -K in leaves and roots. Expression was normalized to that of Actin. Data are means ± SD (n = 3). Means with different letters are significantly different (one-way ANOVA, Duncan, $P \leq 0.05$).

Supplementary Fig. S4

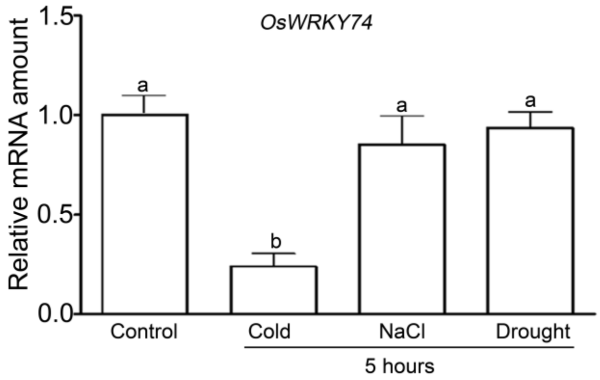

Supplementary Fig. S4. Expression of *OsWRKY74* under cold, NaCl and drought treatments. Expression was normalized to that of Actin. Data are means ± SD (n = 3). Means with different letters are significantly different (one-way ANOVA, Duncan, $P \leq 0.05$).

Supplementary Fig. S5

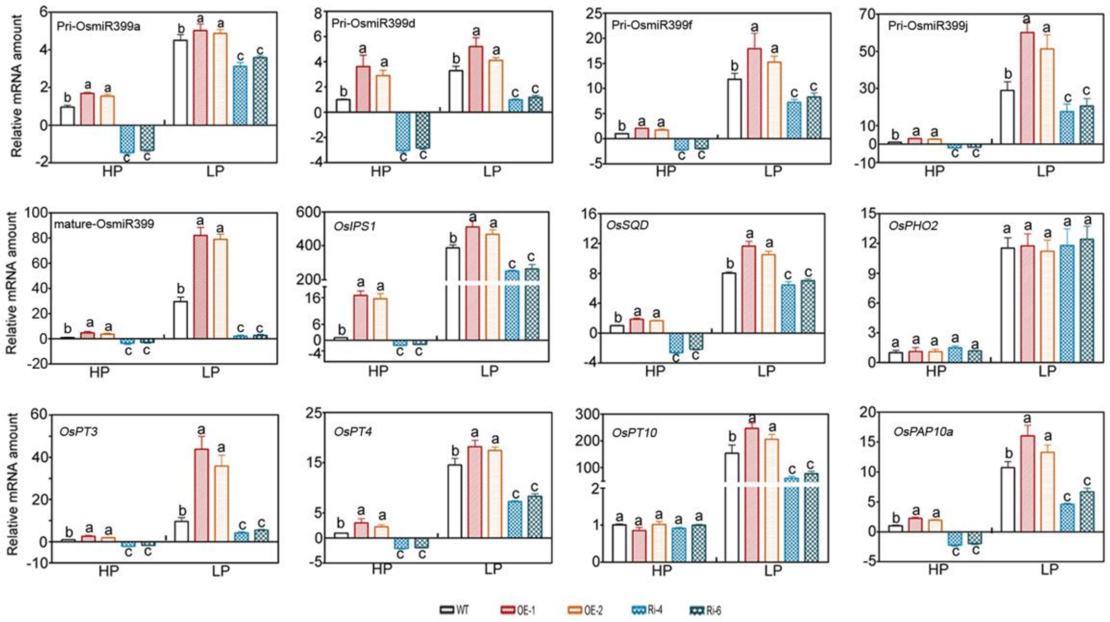

Supplementary Fig. S5. Expression of Pi starvation-induced genes in wild-type (WT) and *OsWRKY74* transgenic plants. Total RNA samples were extracted from shoots of seedling grown in normal nutrient solution for 7 d, followed by treatment with HP or LP medium for 14 d. Expression was normalized to that of *Actin*. Data are means ± SD (n=3). Means with different letters are significantly different (one-way ANOVA, Duncan, $P \leq 0.05$).

Supplemental Table S1 Primers used in Semi-quantitative RT-PCR and Real-time RT-PCR.

Name	Forward primer (5'to3')	Reverse primer (5'to3')	Purpose
OsSQD2	CTGAAAACGGTAATGGATAGG	AACAACAACAGCACGAGC	Real-time PCR
OsPAP10	ATACTGGCAGCCGACGGATGA	GAGGGAGCTGGAGCGGAGAA	Real-time PCR
OsmiR399a	GCTGGAAATGATGCTGGTAGC	CTCCTTTGGCACGAGATCTGT	Real-time PCR
OsmiR399d	GGTGGCCTTTGATAGACCATCA	GCAGGCCGTTTTGGTGAAT	Real-time PCR
OsmiR399f	GGCAGAGGTGATCAGATTGCA	GGCAAATCTCCTTTGGCAGAG	Real-time PCR
OsmiR399j	GGAGCATGTAAGTCTTTTGTAGC	GGCAACTCTCCTTTGGCAGA	Real-time PCR
OsIPS1	CTAAGGTAGGGCAACTTGTATC	TTATTAGAGCAAGGACCGAAAC	Real-time PCR
OsWRKY74Q	GGTCGTTCTTGCCATTCTTGTAC	CTCCATTGCCCTCCATGCT	Real-time PCR
OsPHO2	GGCTATCGGAACTTATGG	AAGAAGGCAGAGGAGGTATC	Real-time PCR
OsActin	GAACTGGTATGGTCAAGGCTG	ACACGGAGCTCGTTGTAGAAG	Real-time PCR
OsWRKY74	TCTCCGACTTGCCTTTCA	AGAACGGGTTGTGCTGAT	Semi-quantitative RT-PCR

Supplemental **Table S2** Sequences of forward and reverse primers and 6-FAM 5' end-labeled probes designed for the 3' UTR of the rice P_i transporter genes and the rice *polyubiquitin 1* (*RubQ1*) gene for quantitative RT-PCR

Name	Forward primer (5'to3')	Reverse primer (5'to3')	Probe (5'to3')
OsPT1	CGCTTCCGTACGAGTGGTAGT	GGTTCTTTCAAATCCAGGGAAA	CACCAATTCTTCAAGAACATATACACACAT
OsPT2	GACGAGACCGCCCAAGAAG	TTTTCAGTCACTCACGTCGAGAC	GATCGCCCAAGCGTGATGTCATAAACAT
OsPT3	TGCGACTGCTGATTCAGTACGT	ACAAATGCCATCAAATATGAACAGA	CATTTGGACACTGCCATTCACATCACACT
OsPT4	TTCTGCTAGTGTACCAAACAAAATTACA	CTAAGTGGCATTTATAATATCAACAGTAACC	TACTAGTCGTCAACCCAGGCAACGCA
OsPT5	TGCTACTGCCCATGACTAGGATT	CCATAGAAGAGATCCAGAGAAGCTGTA	CAACATGGTGAAAATCTGCTGGTTCCCA
OsPT6	CCGCCCCTGCAAACTGTA	CAACTGGCGGTTTCTTCGAT	TAGCGCTGTCGCCGTCTGC
OsPT8	AGAAGGCAAAAGAAATGTGTGTTAAAT	AAAATGTATTCGTGCCAAATTGCT	TCTGCAAGAATTATTCCTCAAGTGGCCAAA
OsPT9	AGAAAAACATAGGCTTGTCATCCTTT	AAAACCTAAGAAGCACTGTAAATAAATCC	CGTTTCCGCTCCTCCTGCCAATC
OsPT10	ATGTCGCCCATCCTTCCA	TCGCTTTCCGACGATGATC	AGTTCACTCACACGGAGACCCGGG
OsPT11	GAGAAGTTCCCTGCTTCAAGCA	TGCATATCCCAGATGAGCGTATC	CGAATGGCAGACATCCATGCACG
OsPT7	GCAAGTCGCTCGAGGAGATG	TGGAATTAACGGGTGGATCAC	CTGGCCGACCTCGTTCTCCCGT
OsPT12	AACGAGATGACGAACACTTGCA	TCCTGTACCTAAAAAGCAAGTACTAACATAGT	TTCCTATCACGCTACAAGTACGAGCTTCGC
OsPT13	TTAGCATAATTCTTTTAGTAGTTAAATAGGAGATG	TGATTTAAGATAAGGATTGAATGCACAT	TTGGTGGCAGATTTCTATCCGTGACACTC
RubQ1	GGGTTCACAAGTCTGCCTATTTG	ACGGGACACGACCAAGGA	CAGACCAGACAACCATAGCTCCATTGGG
